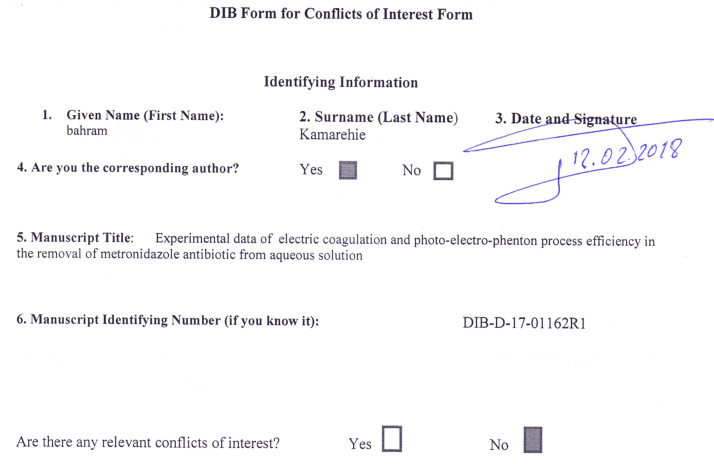# Experimental data of electric coagulation and photo-electro-phenton process efficiency in the removal of metronidazole antibiotic from aqueous solution

**DOI:** 10.1016/j.dib.2018.03.003

**Published:** 2018-03-08

**Authors:** Bahram Kamarehie, Fatemeh Ahmadi, Faria Hafezi, Ali Abbariki, Rouhollah Heydari, Mohammad Amin Karami

**Affiliations:** a Nutrition Health Research Center and Department of Environment Health, School of Health, Lorestan Medical Sciences University, Khorramabad, Iran; b Student Research Committee, lorestan University of Medical Sciences, Khorramabad, Iran; c Razi Herbal Medicines Research Center, Lorestan University of Medical Sciences, Khorramabad, Iran

## Abstract

Pharmaceutical products, particularly antibiotics, due to their cumulative characteristics, undesirable effects and creating drug resistances, as inevitably pollutants, poses a major concern in environmental issues. In recent years, advanced oxidation processes (AOP) have been considerably used for degradation of new and emerging pollutants such as residual medications and resistant compounds in water and wastewater. Present investigation evaluates the removal of metronidazole from aqueous solution by electro coagulation and photoelectrophenton processes. The data will be informative for environmental agencies, pharmaceutical companies and wastewater treatment companies for choosing it as a practical oxidation advance process for treatment of water polluted by resistant material (drugs and pesticides).

**Specifications Table**

**Table:** 

Subject area	Environment
More specific subject are	Wastewater treatment
Type of data	Figure and Table
How data was acquired	High-performance liquid chromatography (HPLC)
Data type	Raw and analyzed
factors Experimental	The study was bench scale that was done in a plexiglass batch reactor, equipped with two electrodes iron (anode) and graphite (cathode) and 4 UV lamps (30 W). Influences of solution pH, time, initial concentration of metronidazole, and electric power in the electric coagulation process and influence of solution pH, time, and initial concentration of metronidazole and electric power, concentration of hydrogen peroxide and intensity of UV radiation in the photo-Electro process on removal efficiency of metronidazole was investigated.
Experimental features	The removal efficiency of metronidazole investigated in Razi research center, Khorramabad
Data source location	Khorramabad, Iran
Data accessibility	Data is with this article.

**Value of the data**

The data may be useful for future researches that aimed in pharmacy wastewater treatment.

This data allows wastewater treatment plants managers and engineers to extend the practical usage of phenton process.

Our data showed that photo-electro-phenton process remove antibiotics from wastewater; an interesting issue for environmentalists who concerned about pharmacy wastewater treatment.

## 1 Data

**Fig. 1:**
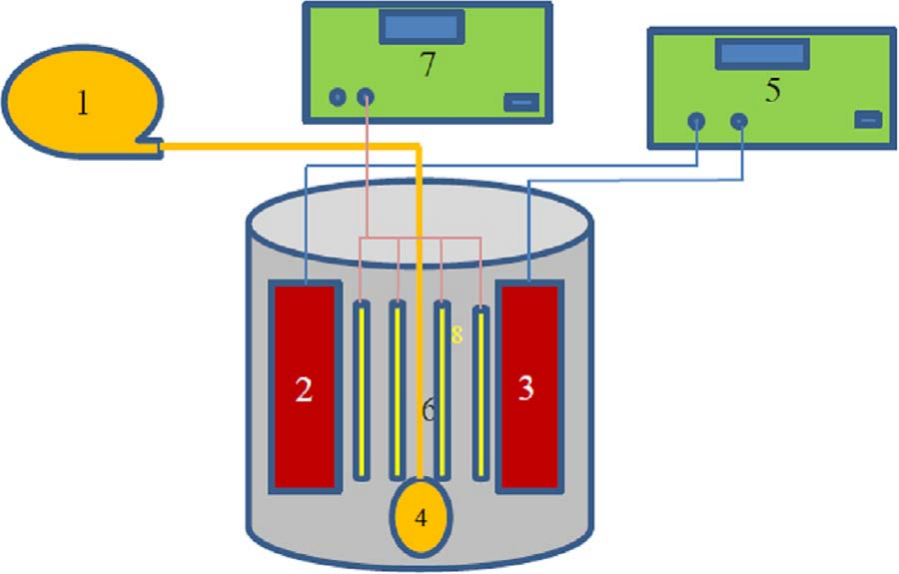
Experimental set up: 1. Air blower, 2- anode, 3- cathode, 4. Ceramic diffuser, 5. Direct supply, 6. Contact reactor, 7. Switching keys, 8. UV lamps.

**Fig. 2:**
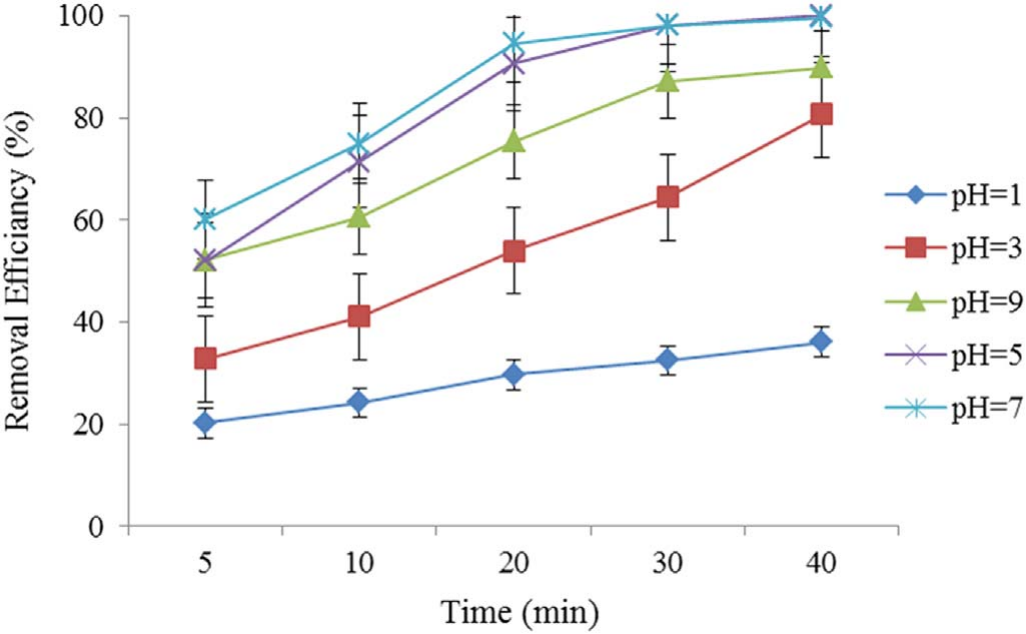
Effect of pH on metronidazole degradation rate (Experimental conditions: metronidazole concentration = 50 mg/L; current density = 25 V; H_2_O_2_ = 0.01 Mol/l, Temperature ~20 °C; UV lamps = 4).

**Fig. 3:**
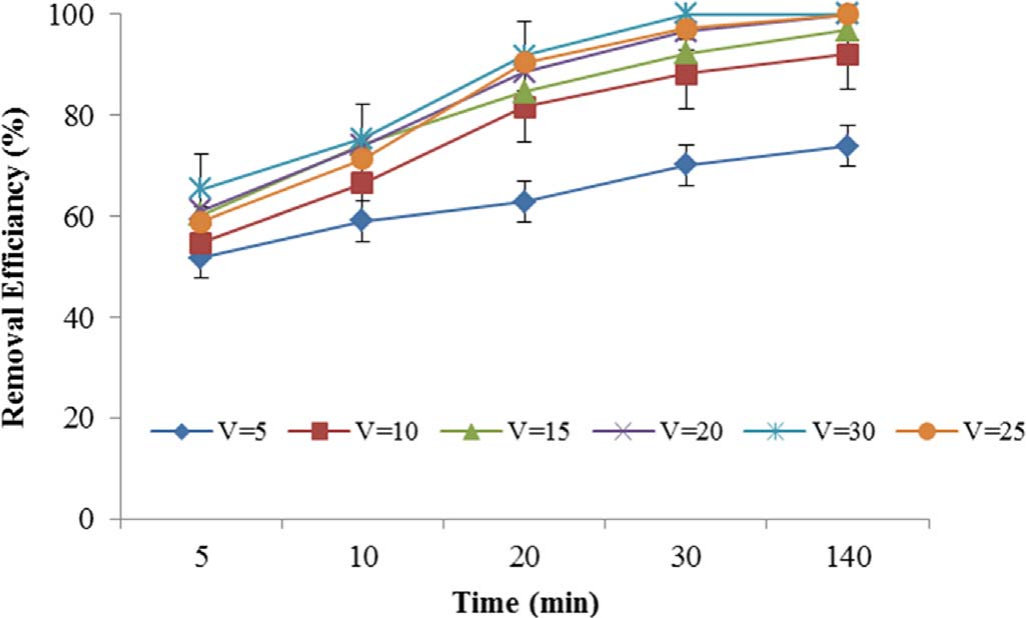
Effect of current density on metronidazole degradation rate (Experimental conditions: metronidazole concentration = 50 mg/L; pH = 7; H_2_O_2_ = 0.01 mol/l, temperature ~20 °C; UV lamps = 4).

**Fig. 4:**
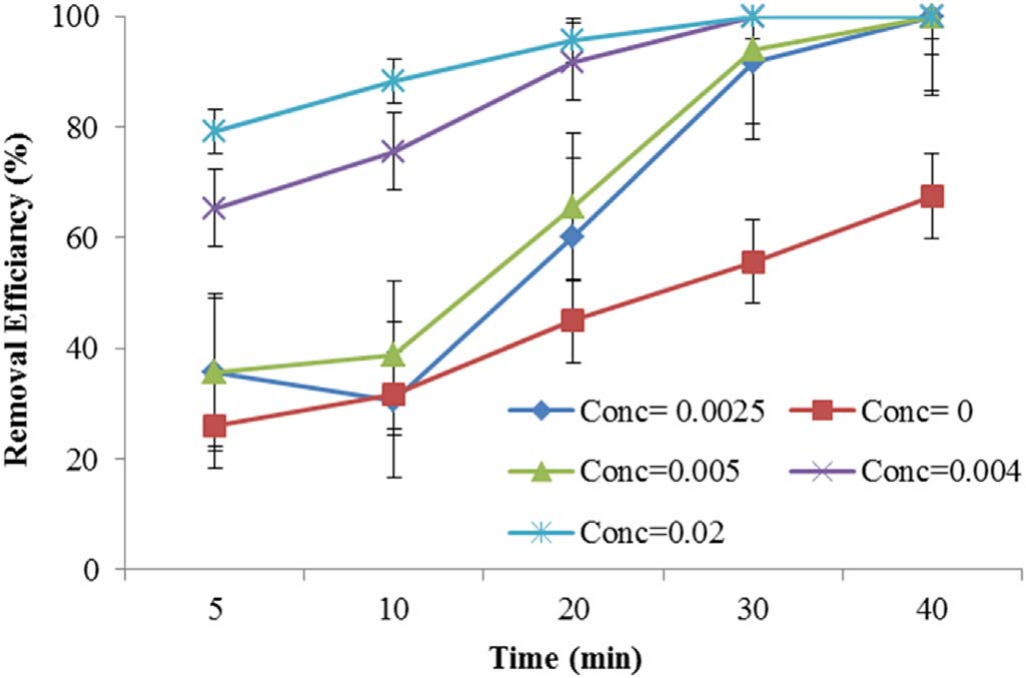
Effect of H_2_O_2_ concentration on metronidazole degradation rate (Experimental conditions: metronidazole concentration = 50 mg/L; pH = 7; current density = 30 V, temperature ~20 °C; UV lamps = 4).

**Fig. 5:**
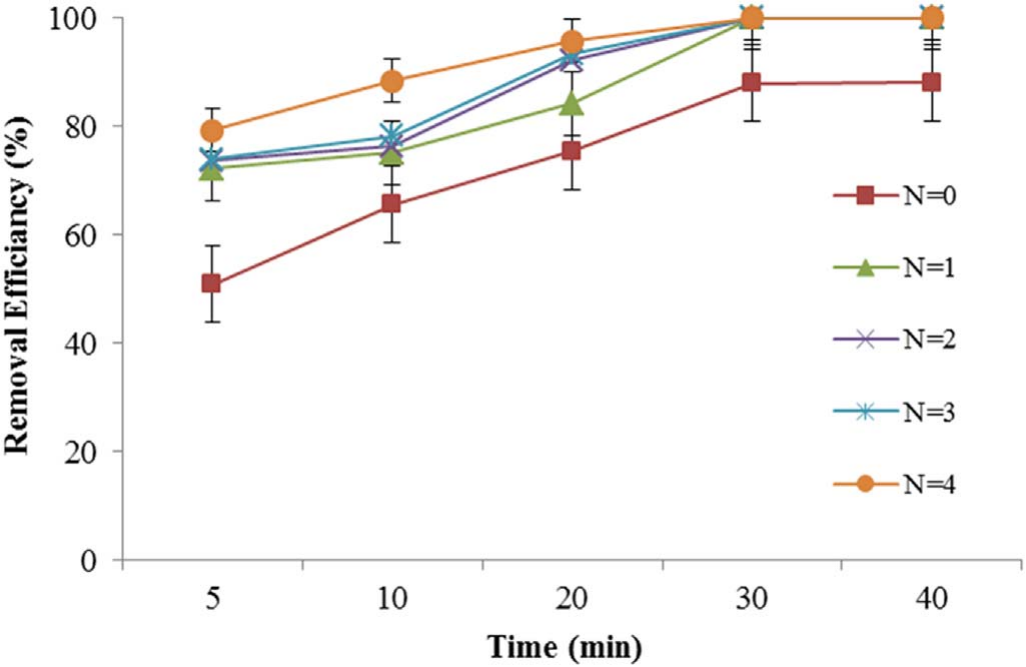
Effect of UV radiation (number lumps) on metronidazole degradation rate (Experimental conditions: metronidazole concentration = 50 mg/L; pH = 7; current density = 30 V, H_2_O_2_ = 0.01 mol/l, temperature ~20 °C).

**Fig. 6:**
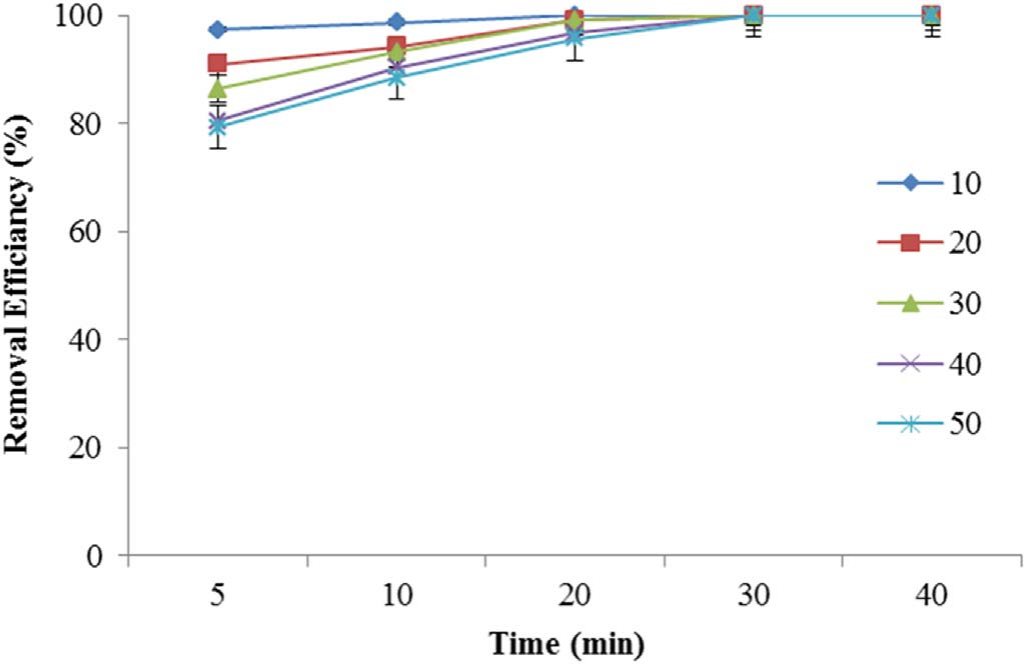
Effect of initial metronidazole concentration on it degradation rate (Experimental conditions: pH = 7; current density = 30 V, H_2_O_2_ = 0.01 mol/l, temperature ~20 °C; UV lamps = 4).

**Fig. 7:**
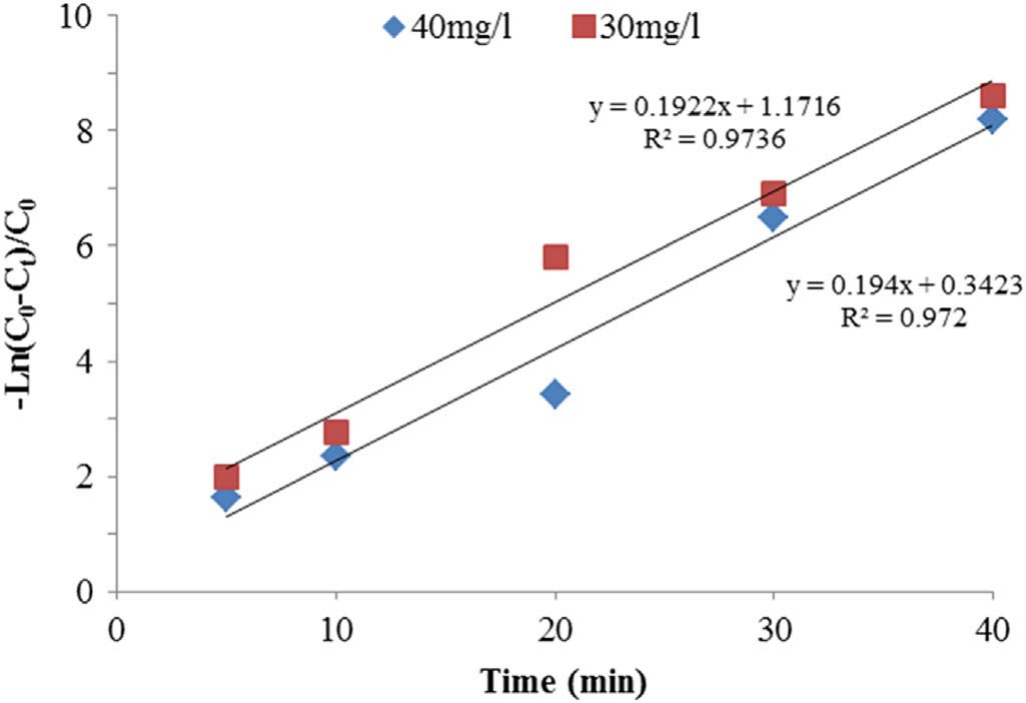
Effect of time on metronidazole removal rate (Experimental conditions: pH = 7; current density = 30 V, H_2_O_2_= 0.01 mol/l, temperature ~20 °C; UV lamps = 4).

This brief dataset describes the use of electro coagulation and photo-electro-phenton process for removing an antibiotic from synthetic wastewater. Table 1 shows physical and chemical properties of metronidazole. The photo-electro-phenton degradation system is given schematically in Fig. 1. The effects of pH, electric current intensity, H_2_O_2_ concentration, UV irradiation, and metronidazole concentration on degradation of metronidazole are presented in Fig. 1, Fig. 2, Fig. 3, Fig. 4, Fig. 5, Fig. 6 respectively (Fig. 7).

**Table 1:** Physical and chemical properties of metronidazole [1], [2].

## 2 Experimental design, materials and methods

In this study, a Plexiglas reactor with working volume of 1 l was used. The rectangular reactor was equipped with two electrodes (iron anode with dimensions (120 × 5 × 120 mm)) and a graphite cathode with dimensions (150 × 120 × 150 mm) and a 5 cm distance to each other. The distance between the electrodes and the reactor wall was 1 cm. An air pump and a ceramic diffuser used for aeration [3]. At each stage, after setting the desired concentration of metronidazole, firstly, the sample pH and the electrical conductivity (1000 ms/l using sodium chloride) adjusted, and then 1 l solution was introduced into the reactor [3], [4], [5]. In addition, before the start of the process, the samples saturated with air blowing for 10 min. Metronidazole concentration measured using high performance liquid chromatography (HPLC) equipped with detector (UV / VIS SCL-10AVP) and column (5 μm, 250 × 4.6 mm) at wavelength 230 nm [6].

## Figures and Tables

**Fig. S1:**